# *catena*-Poly[[[bis­(1,10-phenanthroline-κ^2^ *N*,*N*′)manganese(II)]-μ-2,2′-dithio­dibenzoato-κ^2^ *O*,*O*] methanol hemisolvate monohydrate]

**DOI:** 10.1107/S1600536809033388

**Published:** 2009-09-16

**Authors:** Li-Ming Zhou, Qiang Zhang, Min Hu

**Affiliations:** a Zhengzhou University of Light Industry, Henan Provincial Key Laboratory of Surface & Interface Science, Henan, Zhengzhou 450002, People’s Republic of China

## Abstract

The title complex, {[Mn(C_14_H_8_O_4_S_2_)(C_12_H_8_N_2_)_2_]·0.5CH_3_OH·H_2_O}_*n*_, has a one-dimensional chain structure in which the Mn^II^ atom is six-coordinated by four N atoms from two 1,10-phenanthroline (phen) ligands and two O atoms from two 2,2′-dithio­dibenzoate (*L*) ligands. The *L* ligands adopt a bis­(monodentate) (*syn–anti*) coordination mode and bridge adjacent Mn^II^ centres, generating a chain running along [201]. Adjacent chains are linked into a two-dimensional network, parallel to (10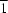), *via* inter­chain C—H⋯π and π–π stacking [centroid–centroid distance = 3.477 (1) Å] inter­actions. The structure also contains numerous hydrogen-bonding interactions, which further link the two-dimensional entities into a three-dimensional supramolecular network.

## Related literature

For related literature on the preparation of functional coordination architectures, see: Robin & Fromm (2006 ▶); Tanaka *et al.* (2008 ▶). For related literature on complexes of 2,2′-dithio­dibenzoic acid, see: Hu *et al.* (2009 ▶); Humphrey *et al.* (2004 ▶); Li *et al.* (2007 ▶); Murugavel *et al.* (2001 ▶); Zhang *et al.* (2006 ▶); Zheng *et al.* (2004 ▶). 
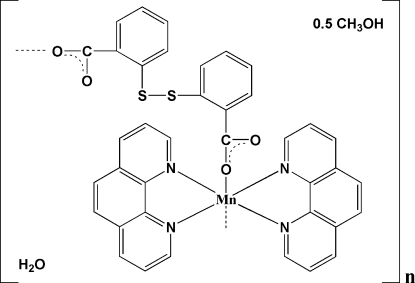


## Experimental

### 

#### Crystal data

[Mn(C_14_H_8_O_4_S_2_)(C_12_H_8_N_2_)_2_]·0.5CH_4_O·H_2_O
*M* *_r_* = 753.71
Monoclinic, 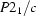

*a* = 12.8267 (11) Å
*b* = 18.3219 (15) Å
*c* = 16.7197 (10) Å
β = 119.989 (4)°
*V* = 3403.2 (5) Å^3^
*Z* = 4
Mo *K*α radiation
μ = 0.56 mm^−1^
*T* = 296 K
0.21 × 0.15 × 0.13 mm

#### Data collection

Bruker SMART CCD area-detector diffractometer
Absorption correction: multi-scan (*SADABS*; Bruker, 2007 ▶) *T*_min_ = 0.891, *T*_max_ = 0.930
24723 measured reflections
5981 independent reflections
4429 reflections with *I* > 2σ(*I*)
*R*_int_ = 0.036

#### Refinement

*R*[*F* ^2^ > 2σ(*F* ^2^)] = 0.040
*wR*(*F* ^2^) = 0.110
*S* = 1.02
5981 reflections
470 parameters
H-atom parameters constrained
Δρ_max_ = 0.44 e Å^−3^
Δρ_min_ = −0.22 e Å^−3^

### 

Data collection: *SMART* (Bruker, 2007 ▶); cell refinement: *SAINT* (Bruker, 2007 ▶); data reduction: *SAINT*; program(s) used to solve structure: *SHELXS97* (Sheldrick, 2008 ▶); program(s) used to refine structure: *SHELXL97* (Sheldrick, 2008 ▶); molecular graphics: *SHELXTL* (Sheldrick, 2008 ▶); software used to prepare material for publication: *SHELXTL*.

## Supplementary Material

Crystal structure: contains datablocks I, global. DOI: 10.1107/S1600536809033388/su2132sup1.cif

Structure factors: contains datablocks I. DOI: 10.1107/S1600536809033388/su2132Isup2.hkl

Additional supplementary materials:  crystallographic information; 3D view; checkCIF report

## Figures and Tables

**Table 1:** Hydrogen-bond geometry (Å, °)

*D*—H⋯*A*	*D*—H	H⋯*A*	*D*⋯*A*	*D*—H⋯*A*
O5—H5⋯O6	0.85	1.94	2.696 (10)	148
O6—H61⋯O4^i^	0.85	1.90	2.746 (4)	177
O6—H62⋯O1	0.85	2.43	2.873 (4)	114
C1—H1*A*⋯O2	0.93	2.47	3.062 (4)	122
C2—H2*A*⋯O4^ii^	0.93	2.47	3.321 (5)	152
C8—H8*A*⋯O5^i^	0.93	2.57	3.438 (13)	156
C21—H21*A*⋯O4^iii^	0.93	2.42	3.291 (5)	155
C27—H27*A*⋯O2	0.93	2.43	2.759 (5)	101
C30—H30*A*⋯S1	0.93	2.58	3.129 (3)	118
C33—H33*A*⋯S2	0.93	2.61	3.161 (3)	119
C36—H36*A*⋯O4	0.93	2.45	2.762 (4)	100
C3—H3*A*⋯*Cg*1^ii^	0.93	2.94	3.795 (39)	153
C6—H6*A*⋯*Cg*2^iv^	0.93	2.85	3.698 (27)	152
C35—H35*A*⋯*Cg*3^iii^	0.93	2.85	3.724 (31)	156

Symmetry codes: (i) 
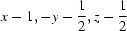
; (ii) 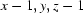; (iii) 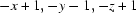; (iv) 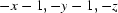. *Cg*1, *Cg*2 and *Cg*3 are the centroids of the C32–C37, C19–C23/N4 and C26–C31 rings, respectively.

**Fig. 1.**
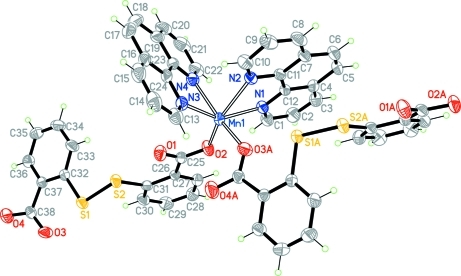
The molecular structure of the title compound, showing the atom-labelling scheme and displacement ellipsoids drawn at the 30% probability level.

**Fig. 2.**
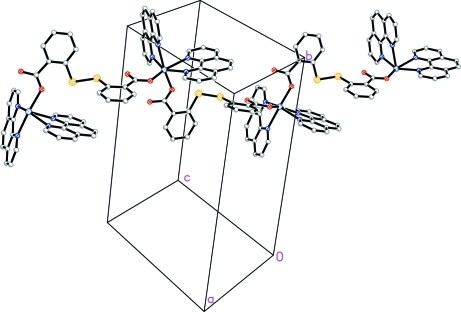
View of the one-dimensional chain of the title compound running along the [201] direction.

**Fig. 3.**
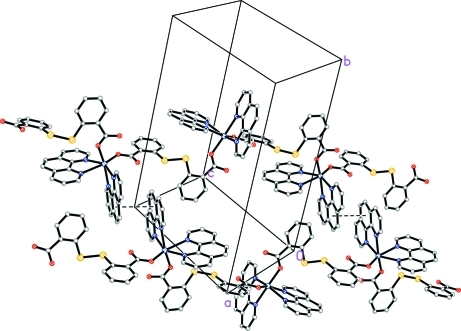
A view, parallel to the (201) plane, of the two-dimensional network of the title compound formed by intermolecular π–π stacking interactions (fine dashed lines) involving the phenyl rings of neighbouring phen ligands.

## supplementary crystallographic information

### Comment

Rational engineering and the preparation of functional coordination architectures with well regulated network structures has attracted increasing interest in recent years (Robin *et al.*, 2006; Tanaka *et al.*, 2008). Among the various ligands used in this field 2,2'-dithiodibenzoic acid (*LH*), a multifunctional ligand containing both carboxylic and thio groups, can potentially afford various coordination modes and diverse coordination architectures (Zhang *et al.*, 2006; Zheng *et al.*, 2004). Many complexes with this ligand show unique structural topologies and interesting properties (Murugavel *et al.*, 2001; Humphrey *et al.*, 2004; Li *et al.*, 2007; Hu *et al.*, 2009). In this work, we have used ligand *LH* to react with a Mn^II^ salt in the presence of 1,10-phenanthroline (phen) as a chelating co-ligand, to obtain the title compound, {[Mn(*L*)(phen)_2_](CH_3_OH)_0.5_(H_2_O)}_n_, a one-dimensional polymer chain.

The asymmetric unit of the title compound is composed of one Mn^II^ atom, one 2,2'-dithiodibenzoate (*L*) ligand, two phen ligands, half a methanol molecule, and one lattice water molecule (Fig. 1). The Mn^II^ center is six-coordinated, in an distorted octahedral geometry, by four nitrogen donors atoms from two phen ligands and two O-atoms from two *L* ligands. The *L* ligands adopt a bis(monodentate)(*syn-anti*) coordination mode to bridge adjacent Mn^II^ centres, generating a one-dimensional chain running along the [201] direction (Fig. 2). In addition, these chains are further arranged into a two-dimensional network, parallel to the (101) plane, by interchain π–π stacking interactions between the phenyl rings of neighbouring phen ligands, with a centroid–centroid separation of 3.477 (1) Å (Fig. 3).

The structure also contains numerous interchain C—H···π (Table 1) interactions between the pyridyl and phenyl rings of the *L* and phen ligands, with an edge-to-face orientation that further links the one-dimensional entities into a two- and then a three-dimensional supramolecular network (Fig. 3).

Footnote for Table 1: C*g*1 is the centroid of ring (C32–C37), C*g*2 is the centroid of ring (C19–C23/N4) and C*g*3 is the centroid of ring (C26–C31).

### Experimental

Caution: Perchlorate salts are dangerous, only small quantites should be used. A solution of 1,10-phenanthroline (phen) (0.05 mmol) and 2,2'-dithiodibenzoic acid (*L*) (0.05 mmol) in CH_3_OH (10 ml) in the presence of excess 2,6-dimethylpyridine (*ca* 0.05 ml for adjusting the pH value of the reaction system to basic conditions) was carefully layered on top of an aqueous solution (15 ml) of Mn(ClO_4_)_2_ (0.1 mmol) in a test tube. Yellow single crystals, suitable for X-ray analysis, appeared at the tube wall after *ca.* one month at rt (Yield ~30% based on *L*). Elemental analysis calculated for (C_38.5_H_28_MnN_4_O_5.5_S_2_): H 3.74 C 61.35 N 7.43%; found: H 3.67, C 61.72, N 7.59%. IR (KBr pellet, cm^-1^): 3417*s*(*br*), 3055*w*, 1600*vs*, 1516*w*, 1423*m*, 1369*vs*, 1342*w*, 1276*w*, 1219*w*, 1143*m*, 1099*m*, 1034*m*, 957*w*, 851*s*, 813*w*, 781*w*, 757*s*, 726*s*, 699*m*, 651*m*, 635*w*, 559*w*, 495*w*, 467*w*, 416*w*.

### Refinement

The methanol and water H-atoms were refined with the O-H distances fixed at 0-H = 0.85 Å and U_iso_(H) = 1.2U_eq_(parent O-atom). The C-bound H-atoms were included in calculated positions and treated as riding atoms: C—H = 0.93 - 0.96 Å with *U*_iso_(H) = 1.2 or 1.5 *U*_eq_(C).

### Crystal data

**Table:**

[Mn(C_14_H_8_O_4_S_2_)(C_12_H_8_N_2_)_2_]·0.5CH_4_O·H_2_O	*F*(000) = 1552
*M**_r_* = 753.71	*D*_x_ = 1.471 Mg m^−^^3^
Monoclinic, *P*2_1_/*c*	Mo *K*α radiation, λ = 0.71073 Å
Hall symbol: -P 2ybc	Cell parameters from 4825 reflections
*a* = 12.8267 (11) Å	θ = 2.5–22.6°
*b* = 18.3219 (15) Å	µ = 0.56 mm^−^^1^
*c* = 16.7197 (10) Å	*T* = 296 K
β = 119.989 (4)°	Block, yellow
*V* = 3403.2 (5) Å^3^	0.21 × 0.15 × 0.13 mm
*Z* = 4	

### Data collection

**Table:**

Bruker SMART CCD area-detector diffractometer	5981 independent reflections
Radiation source: fine-focus sealed tube	4429 reflections with *I* > 2σ(*I*)
graphite	*R*_int_ = 0.036
φ and ω scans	θ_max_ = 25.0°, θ_min_ = 2.5°
Absorption correction: multi-scan (*SADABS*; Bruker, 2007)	*h* = −15→15
*T*_min_ = 0.891, *T*_max_ = 0.930	*k* = −21→21
24723 measured reflections	*l* = −19→19

### Refinement

**Table:**

Refinement on *F*^2^	Primary atom site location: structure-invariant direct methods
Least-squares matrix: full	Secondary atom site location: difference Fourier map
*R*[*F*^2^ > 2σ(*F*^2^)] = 0.040	Hydrogen site location: inferred from neighbouring sites
*wR*(*F*^2^) = 0.110	H-atom parameters constrained
*S* = 1.02	*w* = 1/[σ^2^(*F*_o_^2^) + (0.049*P*)^2^ + 1.4453*P*] where *P* = (*F*_o_^2^ + 2*F*_c_^2^)/3
5981 reflections	(Δ/σ)_max_ < 0.001
470 parameters	Δρ_max_ = 0.44 e Å^−^^3^
0 restraints	Δρ_min_ = −0.22 e Å^−^^3^

### Special details

**Table:**

Geometry. Bond distances, angles etc. have been calculated using the rounded fractional coordinates. All su's are estimated from the variances of the (full) variance-covariance matrix. The cell esds are taken into account in the estimation of distances, angles and torsion angles
Refinement. Refinement of *F*^2^ against ALL reflections. The weighted *R*-factor *wR* and goodness of fit *S* are based on *F*^2^, conventional *R*-factors *R* are based on *F*, with *F* set to zero for negative *F*^2^. The threshold expression of *F*^2^ > σ(*F*^2^) is used only for calculating *R*-factors(gt) *etc*. and is not relevant to the choice of reflections for refinement. *R*-factors based on *F*^2^ are statistically about twice as large as those based on *F*, and *R*- factors based on ALL data will be even larger.

### Fractional atomic coordinates and isotropic or equivalent isotropic displacement parameters (Å^2^)

**Table:**

	*x*	*y*	*z*	*U*_iso_*/*U*_eq_	Occ. (<1)
Mn1	−0.17651 (4)	−0.34583 (2)	0.22410 (3)	0.0455 (1)	
S1	0.49716 (7)	−0.28167 (4)	0.58988 (5)	0.0579 (3)	
S2	0.31804 (6)	−0.29936 (4)	0.49668 (5)	0.0535 (3)	
O1	0.08644 (17)	−0.31820 (12)	0.38122 (14)	0.0644 (8)	
O2	−0.01362 (17)	−0.31846 (12)	0.22892 (14)	0.0646 (8)	
O3	0.73250 (18)	−0.25437 (11)	0.69145 (16)	0.0730 (8)	
O4	0.8689 (2)	−0.32620 (13)	0.79592 (17)	0.0849 (9)	
N1	−0.2507 (2)	−0.37311 (13)	0.07076 (15)	0.0498 (8)	
N2	−0.38325 (19)	−0.39268 (12)	0.15489 (15)	0.0485 (8)	
N3	−0.1817 (2)	−0.36620 (14)	0.35626 (16)	0.0557 (8)	
N4	−0.1148 (2)	−0.46339 (13)	0.26842 (18)	0.0584 (8)	
C1	−0.1851 (3)	−0.36745 (17)	0.0302 (2)	0.0623 (11)	
C2	−0.2331 (3)	−0.3758 (2)	−0.0646 (2)	0.0748 (16)	
C3	−0.3531 (4)	−0.38746 (18)	−0.1191 (2)	0.0738 (13)	
C4	−0.4252 (3)	−0.39356 (16)	−0.07957 (19)	0.0581 (10)	
C5	−0.5516 (3)	−0.40680 (18)	−0.1320 (2)	0.0743 (11)	
C6	−0.6177 (3)	−0.41452 (18)	−0.0915 (2)	0.0753 (11)	
C7	−0.5644 (3)	−0.41030 (15)	0.0066 (2)	0.0579 (10)	
C8	−0.6300 (3)	−0.42031 (17)	0.0518 (3)	0.0723 (13)	
C9	−0.5729 (3)	−0.41726 (18)	0.1452 (3)	0.0721 (14)	
C10	−0.4496 (3)	−0.40331 (17)	0.1942 (2)	0.0621 (11)	
C11	−0.4404 (2)	−0.39659 (14)	0.06130 (19)	0.0471 (9)	
C12	−0.3696 (2)	−0.38726 (13)	0.01737 (18)	0.0457 (9)	
C13	−0.2132 (3)	−0.3183 (2)	0.4003 (2)	0.0697 (11)	
C14	−0.2331 (3)	−0.3376 (3)	0.4723 (2)	0.0876 (18)	
C15	−0.2183 (3)	−0.4079 (3)	0.5000 (3)	0.0922 (16)	
C16	−0.1834 (3)	−0.4606 (2)	0.4572 (2)	0.0744 (13)	
C17	−0.1619 (3)	−0.5365 (3)	0.4840 (3)	0.0984 (18)	
C18	−0.1265 (4)	−0.5835 (3)	0.4415 (3)	0.1012 (18)	
C19	−0.1097 (3)	−0.56169 (19)	0.3673 (3)	0.0795 (14)	
C20	−0.0700 (3)	−0.6082 (2)	0.3213 (4)	0.0980 (16)	
C21	−0.0507 (3)	−0.5831 (2)	0.2538 (3)	0.0930 (18)	
C22	−0.0754 (3)	−0.50955 (18)	0.2286 (3)	0.0729 (14)	
C23	−0.1305 (2)	−0.48828 (16)	0.3376 (2)	0.0596 (10)	
C24	−0.1663 (2)	−0.43674 (18)	0.3844 (2)	0.0578 (10)	
C25	0.0819 (2)	−0.31284 (14)	0.3061 (2)	0.0469 (9)	
C26	0.1968 (2)	−0.29911 (13)	0.30474 (18)	0.0426 (8)	
C27	0.1924 (3)	−0.29218 (15)	0.2204 (2)	0.0541 (10)	
C28	0.2948 (3)	−0.28131 (18)	0.2144 (2)	0.0653 (12)	
C29	0.4040 (3)	−0.27815 (18)	0.2940 (2)	0.0683 (14)	
C30	0.4127 (3)	−0.28476 (16)	0.3790 (2)	0.0573 (11)	
C31	0.3091 (2)	−0.29420 (13)	0.38594 (19)	0.0452 (9)	
C32	0.5624 (2)	−0.37108 (14)	0.62171 (18)	0.0481 (9)	
C33	0.4918 (3)	−0.43378 (17)	0.5900 (2)	0.0722 (11)	
C34	0.5419 (3)	−0.50228 (18)	0.6173 (3)	0.0796 (14)	
C35	0.6624 (3)	−0.50987 (17)	0.6763 (2)	0.0681 (11)	
C36	0.7333 (3)	−0.44861 (16)	0.7096 (2)	0.0569 (11)	
C37	0.6853 (2)	−0.37882 (14)	0.68310 (17)	0.0439 (9)	
C38	0.7696 (2)	−0.31497 (16)	0.7255 (2)	0.0506 (10)	
O5	0.0758 (8)	−0.0482 (5)	0.4927 (9)	0.193 (6)	0.500
C39	0.0897 (12)	−0.0435 (7)	0.5697 (8)	0.164 (7)	0.500
O6	0.0555 (3)	−0.1944 (2)	0.4728 (2)	0.1689 (18)	
H1A	−0.10340	−0.35750	0.06660	0.0750*	
H2A	−0.18350	−0.37340	−0.09040	0.0900*	
H3A	−0.38680	−0.39130	−0.18270	0.0890*	
H5A	−0.58870	−0.41010	−0.19600	0.0890*	
H6A	−0.70000	−0.42280	−0.12760	0.0900*	
H8A	−0.71240	−0.42900	0.01800	0.0870*	
H9A	−0.61540	−0.42440	0.17630	0.0870*	
H10A	−0.41170	−0.40140	0.25820	0.0750*	
H13A	−0.22250	−0.26970	0.38210	0.0840*	
H14A	−0.25610	−0.30260	0.50070	0.1050*	
H15A	−0.23130	−0.42150	0.54790	0.1110*	
H17A	−0.17290	−0.55290	0.53190	0.1190*	
H18A	−0.11240	−0.63180	0.46100	0.1220*	
H20A	−0.05670	−0.65720	0.33760	0.1170*	
H21A	−0.02200	−0.61380	0.22490	0.1120*	
H22A	−0.06350	−0.49230	0.18150	0.0880*	
H27A	0.11820	−0.29490	0.16630	0.0650*	
H28A	0.28970	−0.27620	0.15720	0.0780*	
H29A	0.47340	−0.27140	0.29050	0.0820*	
H30A	0.48790	−0.28300	0.43220	0.0690*	
H33A	0.40940	−0.42940	0.54980	0.0870*	
H34A	0.49320	−0.54350	0.59530	0.0950*	
H35A	0.69640	−0.55610	0.69390	0.0810*	
H36A	0.81530	−0.45390	0.75070	0.0680*	
H5	0.09040	−0.09100	0.48120	0.2320*	0.500
H39A	0.03240	−0.00960	0.56930	0.1960*	0.500
H39B	0.16980	−0.02660	0.61150	0.1960*	0.500
H39C	0.07810	−0.09050	0.58930	0.1960*	0.500
H61	−0.00090	−0.18860	0.41740	0.2030*	
H62	0.11950	−0.21100	0.47610	0.2030*	

### Atomic displacement parameters (Å^2^)

**Table:**

	*U*^11^	*U*^22^	*U*^33^	*U*^12^	*U*^13^	*U*^23^
Mn1	0.0396 (2)	0.0502 (3)	0.0402 (2)	0.0029 (2)	0.0151 (2)	0.0016 (2)
S1	0.0464 (4)	0.0476 (4)	0.0551 (5)	0.0001 (3)	0.0070 (4)	−0.0044 (3)
S2	0.0406 (4)	0.0624 (5)	0.0476 (4)	0.0012 (3)	0.0146 (3)	0.0014 (3)
O1	0.0431 (12)	0.0929 (16)	0.0549 (13)	−0.0011 (11)	0.0228 (10)	0.0116 (11)
O2	0.0371 (11)	0.0890 (15)	0.0533 (13)	−0.0019 (10)	0.0118 (10)	−0.0042 (11)
O3	0.0451 (12)	0.0488 (12)	0.0925 (17)	−0.0049 (10)	0.0100 (12)	0.0039 (11)
O4	0.0589 (15)	0.0882 (17)	0.0722 (16)	−0.0074 (13)	0.0062 (13)	0.0006 (13)
N1	0.0428 (13)	0.0584 (14)	0.0457 (13)	−0.0035 (11)	0.0202 (11)	−0.0033 (11)
N2	0.0422 (13)	0.0495 (13)	0.0518 (14)	−0.0015 (10)	0.0219 (12)	−0.0046 (11)
N3	0.0474 (14)	0.0647 (16)	0.0460 (14)	−0.0040 (12)	0.0167 (12)	0.0039 (12)
N4	0.0401 (13)	0.0572 (15)	0.0635 (16)	0.0029 (11)	0.0152 (12)	0.0023 (13)
C1	0.0587 (19)	0.078 (2)	0.0518 (18)	−0.0071 (16)	0.0289 (16)	−0.0027 (15)
C2	0.084 (3)	0.091 (3)	0.060 (2)	−0.008 (2)	0.044 (2)	0.0003 (19)
C3	0.094 (3)	0.077 (2)	0.0452 (18)	−0.008 (2)	0.031 (2)	−0.0023 (16)
C4	0.065 (2)	0.0525 (17)	0.0433 (16)	−0.0029 (15)	0.0169 (16)	0.0013 (13)
C5	0.068 (2)	0.070 (2)	0.0468 (18)	−0.0133 (18)	0.0000 (18)	−0.0030 (16)
C6	0.0478 (19)	0.073 (2)	0.070 (2)	−0.0080 (16)	0.0030 (18)	−0.0004 (18)
C7	0.0417 (16)	0.0490 (17)	0.067 (2)	−0.0033 (13)	0.0152 (16)	−0.0027 (14)
C8	0.0407 (18)	0.068 (2)	0.095 (3)	−0.0079 (15)	0.0240 (19)	−0.0082 (19)
C9	0.056 (2)	0.075 (2)	0.097 (3)	−0.0094 (17)	0.047 (2)	−0.011 (2)
C10	0.0540 (19)	0.070 (2)	0.068 (2)	−0.0034 (15)	0.0347 (17)	−0.0078 (16)
C11	0.0413 (15)	0.0390 (15)	0.0512 (17)	−0.0002 (11)	0.0157 (13)	−0.0030 (12)
C12	0.0437 (16)	0.0385 (14)	0.0449 (15)	−0.0005 (12)	0.0147 (13)	−0.0001 (12)
C13	0.069 (2)	0.085 (2)	0.0546 (19)	−0.0061 (19)	0.0305 (18)	−0.0088 (17)
C14	0.073 (3)	0.132 (4)	0.058 (2)	−0.011 (2)	0.033 (2)	−0.013 (2)
C15	0.069 (2)	0.150 (4)	0.054 (2)	−0.024 (3)	0.028 (2)	0.011 (3)
C16	0.0443 (18)	0.104 (3)	0.055 (2)	−0.0211 (18)	0.0098 (16)	0.022 (2)
C17	0.056 (2)	0.126 (4)	0.082 (3)	−0.025 (2)	0.011 (2)	0.050 (3)
C18	0.059 (2)	0.089 (3)	0.111 (4)	−0.015 (2)	0.009 (2)	0.049 (3)
C19	0.0430 (19)	0.060 (2)	0.092 (3)	−0.0111 (16)	0.0010 (18)	0.018 (2)
C20	0.052 (2)	0.054 (2)	0.131 (4)	−0.0039 (18)	0.003 (2)	0.005 (2)
C21	0.051 (2)	0.067 (3)	0.125 (4)	0.0073 (18)	0.017 (2)	−0.019 (2)
C22	0.0478 (19)	0.065 (2)	0.090 (3)	0.0061 (16)	0.0225 (18)	−0.0116 (18)
C23	0.0324 (15)	0.0571 (19)	0.063 (2)	−0.0049 (13)	0.0042 (14)	0.0131 (15)
C24	0.0370 (15)	0.072 (2)	0.0475 (17)	−0.0069 (14)	0.0085 (13)	0.0145 (15)
C25	0.0372 (15)	0.0441 (15)	0.0525 (18)	0.0045 (12)	0.0172 (14)	0.0040 (13)
C26	0.0384 (14)	0.0359 (14)	0.0476 (16)	0.0004 (11)	0.0170 (13)	0.0023 (11)
C27	0.0498 (17)	0.0548 (17)	0.0514 (17)	0.0032 (13)	0.0206 (15)	0.0043 (13)
C28	0.070 (2)	0.072 (2)	0.064 (2)	0.0009 (17)	0.0411 (19)	0.0058 (16)
C29	0.059 (2)	0.077 (2)	0.083 (3)	−0.0107 (17)	0.046 (2)	−0.0040 (18)
C30	0.0418 (17)	0.0591 (18)	0.065 (2)	−0.0078 (14)	0.0222 (15)	−0.0044 (15)
C31	0.0406 (15)	0.0368 (14)	0.0529 (16)	−0.0014 (11)	0.0195 (13)	0.0011 (12)
C32	0.0502 (17)	0.0451 (15)	0.0404 (15)	−0.0015 (13)	0.0162 (13)	−0.0009 (12)
C33	0.0515 (19)	0.0539 (19)	0.077 (2)	−0.0056 (15)	0.0064 (17)	−0.0032 (16)
C34	0.072 (2)	0.0468 (19)	0.095 (3)	−0.0109 (17)	0.023 (2)	−0.0010 (18)
C35	0.079 (2)	0.0455 (18)	0.078 (2)	0.0070 (16)	0.038 (2)	0.0061 (16)
C36	0.0528 (18)	0.0576 (18)	0.0582 (19)	0.0072 (15)	0.0261 (15)	0.0029 (15)
C37	0.0466 (16)	0.0476 (15)	0.0393 (14)	−0.0001 (12)	0.0229 (13)	−0.0028 (12)
C38	0.0372 (16)	0.0608 (19)	0.0486 (17)	0.0011 (13)	0.0176 (14)	−0.0057 (14)
O5	0.156 (8)	0.119 (6)	0.362 (16)	−0.035 (5)	0.172 (11)	−0.060 (8)
C39	0.199 (13)	0.188 (13)	0.170 (11)	−0.127 (10)	0.142 (10)	−0.112 (9)
O6	0.121 (3)	0.224 (4)	0.089 (2)	0.080 (3)	−0.002 (2)	−0.051 (2)

### Geometric parameters (Å, °)

**Table:**

Mn1—O2	2.111 (3)	C19—C20	1.403 (6)
Mn1—N1	2.300 (2)	C20—C21	1.351 (7)
Mn1—N2	2.458 (3)	C21—C22	1.400 (5)
Mn1—N3	2.275 (3)	C23—C24	1.440 (4)
Mn1—N4	2.287 (2)	C25—C26	1.506 (4)
Mn1—O3^i^	2.096 (2)	C26—C31	1.404 (4)
S1—S2	2.0574 (12)	C26—C27	1.389 (4)
S1—C32	1.795 (3)	C27—C28	1.381 (6)
S2—C31	1.800 (3)	C28—C29	1.369 (5)
O1—C25	1.232 (4)	C29—C30	1.374 (5)
O2—C25	1.264 (4)	C30—C31	1.401 (5)
O3—C38	1.230 (4)	C32—C37	1.393 (4)
O4—C38	1.246 (4)	C32—C33	1.393 (4)
O5—C39	1.211 (18)	C33—C34	1.380 (5)
O5—H5	0.8500	C34—C35	1.363 (6)
O6—H61	0.8500	C35—C36	1.375 (5)
O6—H62	0.8500	C36—C37	1.392 (4)
N1—C1	1.323 (5)	C37—C38	1.508 (4)
N1—C12	1.352 (4)	C1—H1A	0.9300
N2—C11	1.358 (4)	C2—H2A	0.9300
N2—C10	1.324 (5)	C3—H3A	0.9300
N3—C13	1.332 (5)	C5—H5A	0.9300
N3—C24	1.356 (4)	C6—H6A	0.9300
N4—C22	1.323 (5)	C8—H8A	0.9300
N4—C23	1.349 (4)	C9—H9A	0.9300
C1—C2	1.393 (4)	C10—H10A	0.9300
C2—C3	1.358 (6)	C13—H13A	0.9300
C3—C4	1.384 (6)	C14—H14A	0.9300
C4—C5	1.427 (5)	C15—H15A	0.9300
C4—C12	1.412 (4)	C17—H17A	0.9300
C5—C6	1.331 (6)	C18—H18A	0.9300
C6—C7	1.429 (4)	C20—H20A	0.9300
C7—C11	1.405 (5)	C21—H21A	0.9300
C7—C8	1.396 (6)	C22—H22A	0.9300
C8—C9	1.355 (6)	C27—H27A	0.9300
C9—C10	1.394 (6)	C28—H28A	0.9300
C11—C12	1.435 (4)	C29—H29A	0.9300
C13—C14	1.395 (5)	C30—H30A	0.9300
C14—C15	1.350 (8)	C33—H33A	0.9300
C15—C16	1.402 (6)	C34—H34A	0.9300
C16—C17	1.445 (7)	C35—H35A	0.9300
C16—C24	1.410 (5)	C36—H36A	0.9300
C17—C18	1.332 (7)	C39—H39C	0.9600
C18—C19	1.419 (7)	C39—H39A	0.9600
C19—C23	1.412 (5)	C39—H39B	0.9600
			
O2—Mn1—N1	86.81 (9)	C25—C26—C27	119.2 (3)
O2—Mn1—N2	156.27 (8)	C25—C26—C31	122.3 (2)
O2—Mn1—N3	120.73 (9)	C26—C27—C28	122.0 (3)
O2—Mn1—N4	92.05 (10)	C27—C28—C29	118.9 (3)
O2—Mn1—O3^i^	102.33 (9)	C28—C29—C30	121.1 (4)
N1—Mn1—N2	69.60 (9)	C29—C30—C31	120.4 (3)
N1—Mn1—N3	148.28 (10)	S2—C31—C26	119.9 (2)
N1—Mn1—N4	92.29 (9)	S2—C31—C30	121.1 (2)
O3^i^—Mn1—N1	91.93 (9)	C26—C31—C30	119.0 (3)
N2—Mn1—N3	81.46 (9)	S1—C32—C37	120.0 (2)
N2—Mn1—N4	86.61 (9)	C33—C32—C37	118.5 (3)
O3^i^—Mn1—N2	81.61 (9)	S1—C32—C33	121.5 (2)
N3—Mn1—N4	72.79 (10)	C32—C33—C34	121.2 (3)
O3^i^—Mn1—N3	96.54 (10)	C33—C34—C35	120.3 (3)
O3^i^—Mn1—N4	165.22 (11)	C34—C35—C36	119.4 (3)
S2—S1—C32	105.04 (10)	C35—C36—C37	121.5 (3)
S1—S2—C31	104.11 (11)	C36—C37—C38	117.6 (3)
Mn1—O2—C25	119.6 (2)	C32—C37—C36	119.1 (3)
Mn1^ii^—O3—C38	129.3 (2)	C32—C37—C38	123.3 (2)
C39—O5—H5	112.00	O3—C38—C37	117.5 (3)
H61—O6—H62	113.00	O3—C38—O4	124.3 (3)
Mn1—N1—C12	118.6 (2)	O4—C38—C37	118.1 (3)
C1—N1—C12	118.2 (2)	C2—C1—H1A	119.00
Mn1—N1—C1	122.7 (2)	N1—C1—H1A	119.00
Mn1—N2—C11	113.26 (19)	C1—C2—H2A	120.00
C10—N2—C11	117.0 (3)	C3—C2—H2A	120.00
Mn1—N2—C10	128.87 (19)	C4—C3—H3A	120.00
C13—N3—C24	117.9 (3)	C2—C3—H3A	120.00
Mn1—N3—C13	126.4 (2)	C4—C5—H5A	119.00
Mn1—N3—C24	115.07 (19)	C6—C5—H5A	119.00
C22—N4—C23	118.3 (3)	C5—C6—H6A	119.00
Mn1—N4—C22	126.6 (2)	C7—C6—H6A	119.00
Mn1—N4—C23	114.9 (2)	C7—C8—H8A	120.00
N1—C1—C2	122.9 (3)	C9—C8—H8A	120.00
C1—C2—C3	119.3 (4)	C10—C9—H9A	120.00
C2—C3—C4	119.8 (3)	C8—C9—H9A	121.00
C3—C4—C12	117.8 (3)	N2—C10—H10A	118.00
C3—C4—C5	123.1 (3)	C9—C10—H10A	118.00
C5—C4—C12	119.1 (3)	N3—C13—H13A	118.00
C4—C5—C6	121.5 (3)	C14—C13—H13A	118.00
C5—C6—C7	121.1 (3)	C15—C14—H14A	121.00
C6—C7—C8	122.8 (4)	C13—C14—H14A	121.00
C6—C7—C11	119.5 (3)	C14—C15—H15A	120.00
C8—C7—C11	117.6 (3)	C16—C15—H15A	120.00
C7—C8—C9	119.6 (4)	C16—C17—H17A	119.00
C8—C9—C10	119.1 (4)	C18—C17—H17A	119.00
N2—C10—C9	123.9 (3)	C19—C18—H18A	119.00
C7—C11—C12	119.2 (3)	C17—C18—H18A	119.00
N2—C11—C7	122.9 (3)	C19—C20—H20A	119.00
N2—C11—C12	117.9 (3)	C21—C20—H20A	119.00
C4—C12—C11	119.5 (3)	C22—C21—H21A	121.00
N1—C12—C4	122.0 (3)	C20—C21—H21A	121.00
N1—C12—C11	118.6 (2)	N4—C22—H22A	118.00
N3—C13—C14	123.2 (4)	C21—C22—H22A	118.00
C13—C14—C15	118.9 (4)	C26—C27—H27A	119.00
C14—C15—C16	120.5 (4)	C28—C27—H27A	119.00
C17—C16—C24	118.6 (3)	C29—C28—H28A	120.00
C15—C16—C17	124.4 (4)	C27—C28—H28A	121.00
C15—C16—C24	117.0 (3)	C28—C29—H29A	119.00
C16—C17—C18	121.3 (4)	C30—C29—H29A	119.00
C17—C18—C19	121.7 (5)	C31—C30—H30A	120.00
C20—C19—C23	116.3 (4)	C29—C30—H30A	120.00
C18—C19—C20	124.3 (4)	C34—C33—H33A	119.00
C18—C19—C23	119.4 (4)	C32—C33—H33A	119.00
C19—C20—C21	121.3 (4)	C33—C34—H34A	120.00
C20—C21—C22	118.1 (4)	C35—C34—H34A	120.00
N4—C22—C21	123.4 (4)	C34—C35—H35A	120.00
N4—C23—C24	118.0 (3)	C36—C35—H35A	120.00
N4—C23—C19	122.7 (3)	C37—C36—H36A	119.00
C19—C23—C24	119.3 (3)	C35—C36—H36A	119.00
N3—C24—C23	117.9 (3)	O5—C39—H39B	109.00
C16—C24—C23	119.7 (3)	O5—C39—H39C	109.00
N3—C24—C16	122.5 (3)	O5—C39—H39A	109.00
O2—C25—C26	117.1 (3)	H39A—C39—H39C	110.00
O1—C25—O2	124.2 (3)	H39B—C39—H39C	109.00
O1—C25—C26	118.7 (3)	H39A—C39—H39B	109.00
C27—C26—C31	118.5 (3)		
			
N1—Mn1—O2—C25	−163.8 (2)	N1—C1—C2—C3	2.7 (5)
N2—Mn1—O2—C25	−157.9 (2)	C1—C2—C3—C4	−2.4 (5)
N3—Mn1—O2—C25	−0.5 (2)	C2—C3—C4—C5	−179.1 (3)
N4—Mn1—O2—C25	−71.6 (2)	C2—C3—C4—C12	0.0 (5)
O3^i^—Mn1—O2—C25	104.9 (2)	C5—C4—C12—C11	2.0 (4)
O2—Mn1—N1—C1	1.1 (2)	C3—C4—C5—C6	178.0 (3)
O2—Mn1—N1—C12	−171.0 (2)	C3—C4—C12—C11	−177.2 (3)
N2—Mn1—N1—C1	−176.4 (3)	C5—C4—C12—N1	−178.5 (3)
N2—Mn1—N1—C12	11.53 (18)	C12—C4—C5—C6	−1.1 (5)
N3—Mn1—N1—C1	−151.0 (2)	C3—C4—C12—N1	2.4 (4)
N3—Mn1—N1—C12	37.0 (3)	C4—C5—C6—C7	−0.4 (5)
N4—Mn1—N1—C1	−90.9 (2)	C5—C6—C7—C11	1.0 (5)
N4—Mn1—N1—C12	97.1 (2)	C5—C6—C7—C8	−178.0 (3)
O3^i^—Mn1—N1—C1	103.3 (2)	C6—C7—C8—C9	178.4 (3)
O3^i^—Mn1—N1—C12	−68.8 (2)	C8—C7—C11—C12	178.9 (3)
O2—Mn1—N2—C10	173.1 (2)	C6—C7—C11—N2	−179.2 (3)
O2—Mn1—N2—C11	−18.3 (3)	C8—C7—C11—N2	−0.1 (4)
N1—Mn1—N2—C10	179.4 (3)	C6—C7—C11—C12	−0.2 (4)
N1—Mn1—N2—C11	−11.92 (17)	C11—C7—C8—C9	−0.6 (4)
N3—Mn1—N2—C10	12.6 (2)	C7—C8—C9—C10	0.7 (5)
N3—Mn1—N2—C11	−178.72 (19)	C8—C9—C10—N2	0.0 (5)
N4—Mn1—N2—C10	85.7 (3)	C7—C11—C12—C4	−1.3 (4)
N4—Mn1—N2—C11	−105.63 (19)	C7—C11—C12—N1	179.1 (2)
O3^i^—Mn1—N2—C10	−85.3 (3)	N2—C11—C12—C4	177.8 (2)
O3^i^—Mn1—N2—C11	83.31 (19)	N2—C11—C12—N1	−1.8 (4)
O2—Mn1—N3—C13	97.3 (3)	N3—C13—C14—C15	1.0 (6)
O2—Mn1—N3—C24	−92.0 (2)	C13—C14—C15—C16	0.1 (6)
N1—Mn1—N3—C13	−115.8 (3)	C14—C15—C16—C17	178.1 (4)
N1—Mn1—N3—C24	55.0 (3)	C14—C15—C16—C24	−0.7 (6)
N2—Mn1—N3—C13	−91.8 (3)	C15—C16—C17—C18	−178.6 (4)
N2—Mn1—N3—C24	79.0 (2)	C15—C16—C24—N3	0.4 (5)
N4—Mn1—N3—C13	179.2 (3)	C15—C16—C24—C23	−180.0 (3)
N4—Mn1—N3—C24	−10.1 (2)	C17—C16—C24—C23	1.2 (5)
O3^i^—Mn1—N3—C13	−11.3 (3)	C17—C16—C24—N3	−178.4 (3)
O3^i^—Mn1—N3—C24	159.5 (2)	C24—C16—C17—C18	0.1 (6)
O2—Mn1—N4—C22	−54.1 (3)	C16—C17—C18—C19	−0.8 (7)
O2—Mn1—N4—C23	131.6 (2)	C17—C18—C19—C20	178.5 (5)
N1—Mn1—N4—C22	32.8 (3)	C17—C18—C19—C23	0.2 (7)
N1—Mn1—N4—C23	−141.5 (2)	C18—C19—C20—C21	−177.0 (5)
N2—Mn1—N4—C22	102.2 (3)	C18—C19—C23—N4	179.1 (4)
N2—Mn1—N4—C23	−72.1 (2)	C23—C19—C20—C21	1.3 (7)
N3—Mn1—N4—C22	−175.7 (3)	C20—C19—C23—C24	−177.3 (4)
N3—Mn1—N4—C23	10.0 (2)	C18—C19—C23—C24	1.1 (6)
O2—Mn1—O3^i^—C38^i^	−47.1 (3)	C20—C19—C23—N4	0.7 (6)
N1—Mn1—O3^i^—C38^i^	−134.3 (3)	C19—C20—C21—C22	−2.1 (7)
N2—Mn1—O3^i^—C38^i^	156.7 (3)	C20—C21—C22—N4	1.1 (7)
N3—Mn1—O3^i^—C38^i^	76.4 (3)	C19—C23—C24—N3	177.9 (3)
C32—S1—S2—C31	−94.30 (13)	C19—C23—C24—C16	−1.8 (5)
S2—S1—C32—C37	178.4 (2)	N4—C23—C24—C16	−179.9 (3)
S2—S1—C32—C33	−5.0 (3)	N4—C23—C24—N3	−0.2 (4)
S1—S2—C31—C26	−175.86 (18)	O1—C25—C26—C31	1.8 (4)
S1—S2—C31—C30	3.7 (2)	O2—C25—C26—C27	1.0 (4)
Mn1—O2—C25—C26	175.25 (16)	O2—C25—C26—C31	−177.9 (2)
Mn1—O2—C25—O1	−4.4 (4)	O1—C25—C26—C27	−179.3 (3)
Mn1^ii^—O3—C38—C37	−175.79 (19)	C25—C26—C31—S2	−3.5 (3)
Mn1^ii^—O3—C38—O4	8.9 (5)	C25—C26—C31—C30	177.0 (2)
C1—N1—C12—C4	−2.2 (4)	C31—C26—C27—C28	0.6 (4)
C12—N1—C1—C2	−0.4 (4)	C25—C26—C27—C28	−178.4 (3)
Mn1—N1—C12—C4	170.3 (2)	C27—C26—C31—S2	177.6 (2)
Mn1—N1—C12—C11	−10.2 (3)	C27—C26—C31—C30	−1.9 (4)
C1—N1—C12—C11	177.4 (3)	C26—C27—C28—C29	0.8 (5)
Mn1—N1—C1—C2	−172.5 (2)	C27—C28—C29—C30	−0.8 (5)
Mn1—N2—C11—C12	11.6 (3)	C28—C29—C30—C31	−0.7 (5)
Mn1—N2—C10—C9	167.6 (2)	C29—C30—C31—C26	2.0 (4)
C11—N2—C10—C9	−0.7 (4)	C29—C30—C31—S2	−177.5 (2)
C10—N2—C11—C12	−178.3 (2)	S1—C32—C37—C36	177.6 (2)
Mn1—N2—C11—C7	−169.3 (2)	S1—C32—C37—C38	−0.2 (4)
C10—N2—C11—C7	0.8 (4)	C33—C32—C37—C36	0.8 (4)
Mn1—N3—C24—C16	−171.0 (3)	C33—C32—C37—C38	−177.0 (3)
C13—N3—C24—C23	−179.1 (3)	C37—C32—C33—C34	−1.0 (5)
Mn1—N3—C24—C23	9.3 (3)	S1—C32—C33—C34	−177.7 (3)
Mn1—N3—C13—C14	169.3 (3)	C32—C33—C34—C35	0.0 (6)
C13—N3—C24—C16	0.6 (5)	C33—C34—C35—C36	1.1 (6)
C24—N3—C13—C14	−1.3 (5)	C34—C35—C36—C37	−1.2 (5)
C22—N4—C23—C24	176.3 (3)	C35—C36—C37—C38	178.2 (3)
Mn1—N4—C23—C19	173.1 (3)	C35—C36—C37—C32	0.3 (5)
Mn1—N4—C22—C21	−173.3 (3)	C32—C37—C38—O3	−13.8 (4)
Mn1—N4—C23—C24	−8.9 (4)	C36—C37—C38—O4	−16.1 (4)
C23—N4—C22—C21	0.9 (6)	C32—C37—C38—O4	161.8 (3)
C22—N4—C23—C19	−1.7 (5)	C36—C37—C38—O3	168.4 (3)

Symmetry codes: (i) *x*−1, −*y*−1/2, *z*−1/2; (ii) *x*+1, −*y*−1/2, *z*+1/2.

### Hydrogen-bond geometry (Å, °)

**Table:**

*D*—H···*A*	*D*—H	H···*A*	*D*···*A*	*D*—H···*A*
O5—H5···O6	0.85	1.94	2.696 (10)	148
O6—H61···O4^i^	0.85	1.90	2.746 (4)	177
O6—H62···O1	0.85	2.43	2.873 (4)	114
C1—H1A···O2	0.93	2.47	3.062 (4)	122
C2—H2A···O4^iii^	0.93	2.47	3.321 (5)	152
C8—H8A···O5^i^	0.93	2.57	3.438 (13)	156
C21—H21A···O4^iv^	0.93	2.42	3.291 (5)	155
C27—H27A···O2	0.93	2.43	2.759 (5)	101
C30—H30A···S1	0.93	2.58	3.129 (3)	118
C33—H33A···S2	0.93	2.61	3.161 (3)	119
C36—H36A···O4	0.93	2.45	2.762 (4)	100
C3—H3A···Cg1^iii^	0.93	2.94	3.795 (39)	153
C6—H6A···Cg2^v^	0.93	2.85	3.698 (27)	152
C35—H35A···Cg3^iv^	0.93	2.85	3.724 (31)	156

Symmetry codes: (i) *x*−1, −*y*−1/2, *z*−1/2; (iii) *x*−1, *y*, *z*−1; (iv) −*x*+1, −*y*−1, −*z*+1; (v) −*x*−1, −*y*−1, −*z*.
